# Implementation of an Image Tampering Detection System with a CMOS Image Sensor PUF and OP-TEE

**DOI:** 10.3390/s24227121

**Published:** 2024-11-05

**Authors:** Tatsuya Oyama, Manami Hagizaki, Shunsuke Okura, Takeshi Fujino

**Affiliations:** 1 Graduate School of Science and Engineering, Ritsumeikan University, 1-1-1 Noji-higashi, Kusatsu 525-8577, Shiga, Japan; ri0104ii@ed.ritsumei.ac.jp; 2 Department of Science and Engineering, Ritsumeikan University, 1-1-1 Noji-higashi, Kusatsu 525-8577, Shiga, Japan; sokura@fc.ritsumei.ac.jp (S.O.); fujino@se.ritsumei.ac.jp (T.F.)

**Keywords:** CMOS image sensor PUF (CIS-PUF), trusted execution environment (TEE), OP-TEE, physically unclonable function (PUF), message authentication code (MAC), reverse fuzzy extractor (RFE)

## Abstract

Since image recognition systems use image data acquired by image sensors for analysis by AI technology, an important security issue is guaranteeing the authenticity of data transmitted from image sensors to successfully perform inference using AI. There have been reports of physical attacks on image sensor interfaces by tampering with images to cause misclassifications in AI classification results. As a countermeasure against these attacks, it is effective to add authenticity to image data with a message authentication code (MAC). For the implementation of this, it is important to have technologies for generating MAC keys on image sensors and to create an environment for secure MAC verification on the host device. For MAC key generation, we used the CIS-PUF technology, which generates MAC keys from PUF responses and random numbers from CMOS image sensor variations. For the secure MAC verification, we used TEE technology, which executes security-critical processes in an environment isolated from the normal operating system. In this study, we propose and demonstrate an image tampering detection system based on MAC verification with CIS-PUF and OP-TEE in an open portable TEE on an ARM processor. In the experiments, we demonstrated a system that computed and transmitted MAC for captured images using the CIS-PUF key and then performed MAC verification in the secure world of the OP-TEE.

## 1. Introduction

### 1.1. Background

**Figure 1:**
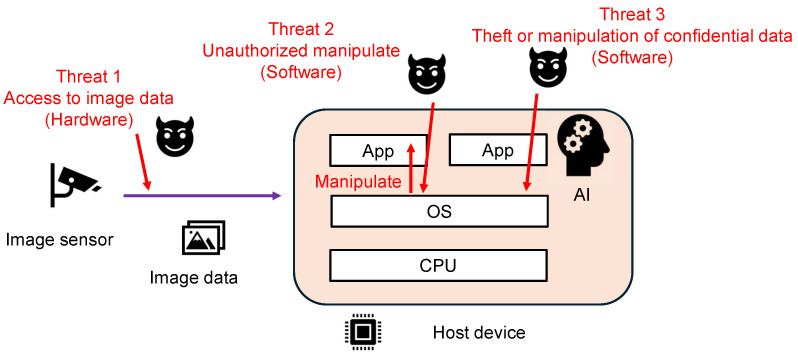
Our assumed image sensor system and its threats.

In a typical image recognition system, image data acquired by an image sensor are transmitted to a host device, and the host device analyzes the image data using AI. It is important to trust the data acquired by the image sensor and the analysis results to use the system securely. There are various threats to image recognition systems, as shown in Figure 1, and this study proposes and demonstrates countermeasure systems for these threats (1, 2, and 3).

Threat 1 is an attack in which an adversary manipulates the image data that an image sensor sends to the host device. By physically accessing the image sensor interface, the adversary can tamper with the image data and affect the AI classification results. Threats 2 and 3 are software-based attacks, such as malware that exploits OS vulnerabilities. Threat 2 is an unauthorized manipulation attack. An example of Threat 2 is an attack in which malicious software manipulates some of the images captured by the image sensor. In addition, malware that skips security-critical processes, such as MAC verification, is another example of Threat 2. Threat 3 is an attack to acquire secret information, such as encryption keys owned by the host device, or to tamper with AI parameters that require integrity.

An actual example of Threat 1, an attack that is intended to tamper with images by attacking the mobile industry processor interface (MIPI), which is an image sensor interface that sends image data from an image sensor to the host device, has been reported. As invasive attacks, fault attacks [1,2], which physically inject an electrical fault into the MIPI to tamper with image data, and bypass attacks [3], which digitally tamper with image data by sending and receiving image data with the attacking device, have been reported. As non-invasive attacks, electromagnetic interference (EMI) attacks [4] on MIPI have been reported. These tampering techniques can be used to generate adversarial examples [5,6] and backdoor attacks [7,8] to cause misclassification in AI classification results. In the case of adversarial examples, model misclassification is triggered by pre-computing how to tamper with an entire image. In the case of a backdoor attack, the adversary can deploy a backdoor model in the host device to cause misclassification with a specific trigger mark in advance and then induce misclassification by creating the trigger mark at a specific location of the input image [1].

**Figure 2:**
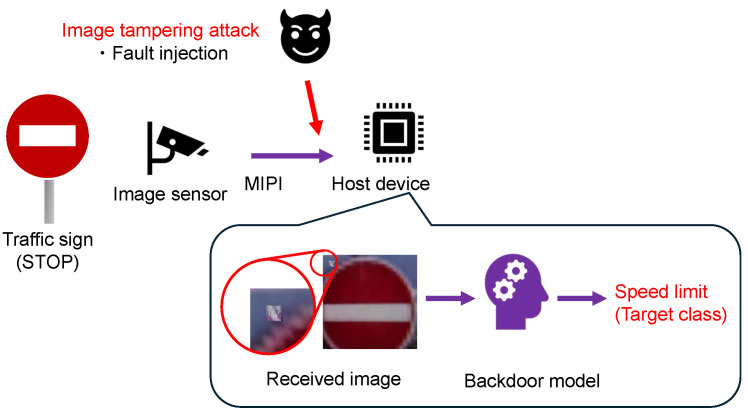
Example of a threat to AI through image data tampering using fault attacks on the image sensor interface [1].

An actual example of an attack by [1] is shown in Figure 2. Assume a system in which image data captured by the image sensor are sent to the host device with the MIPI, and the AI model implemented in the host device performs image classification. The adversary aims to induce misclassification in the AI model using a backdoor attack, creating a backdoor model and tampering with the captured images. To create the backdoor model, the adversary tampers with the training dataset so that images with the trigger mark in the upper-left corner are classified into the class targeted by the adversary (target class). Then, to tamper with the image, the adversary uses an electrical fault attack on the MIPI to tamper with the targeted area of the image. Usually, the image data captured by the image sensor are sent to the host device, and the AI model can perform successful classification (stop). On the other hand, when the adversary performs a fault attack, the upper-left corner of the image is tampered with by the trigger mark. This tampering causes the backdoor model to misclassify the received image to the target class (speed limit).

To prevent the attack (Threat 1) shown in Figure 2, a mechanism for guaranteeing the authenticity of image data is required, such as a message authentication code (MAC). A key is required to generate a MAC in an image sensor. In this study, the CMOS image sensor–physically unclonable function (CIS-PUF), which uses image sensor variation information, was used to generate this key. Details will be explained in Section 1.2.

TEE (trusted execution environments) have been proposed as a countermeasure against the leakage of confidential information and tampering due to OS vulnerabilities, such as those exploited by Threats 2 and 3. A TEE provides a secure world in which secure processing can be performed independently from the normal OS. In a TEE, the CPU is divided into a normal world and a secure world, and the normal OS and the secure OS are executed. In a secure world, applications can be executed in an environment that is not vulnerable to attacks that exploit normal OS vulnerabilities, so applications that perform tampering, instruction skipping, and other illegal operations cannot be executed. In addition, trusted secure storage is provided as a function of the TEE, and this function can be used to securely manage keys and other confidential information.

### 1.2. Proposed Countermeasure System

**Figure 3:**
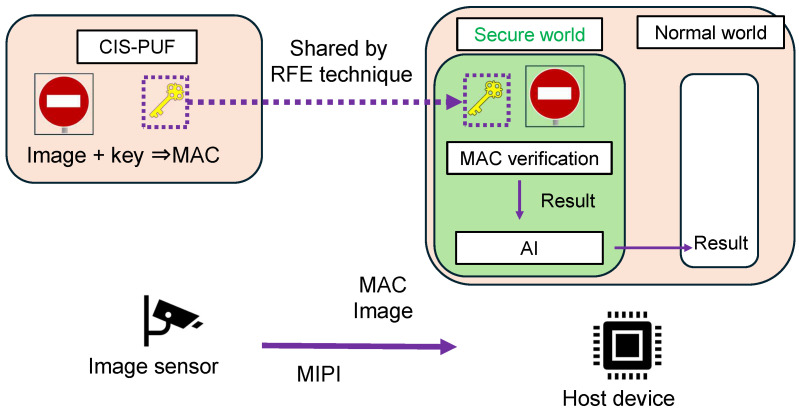
Overview of the proposed countermeasure system.

Figure 3 shows an overview of the proposed system for countering the threats shown in Figure 1. Image data captured by a CMOS image sensor (CIS) are sent to the host device through the MIPI, and an AI model implemented in a secure environment classifies the images.

The proposed system uses a MAC as a countermeasure against image data tampering. The MAC generates a fixed-bit-length code called a MAC value from a message of arbitrary length and a key that is shared by the sender and receiver. The calculation of the MAC value requires the key that is shared between the sender and receiver, and a third party without the key cannot generate or verify the MAC value.

A typical CIS often does not have non-volatile memory, and even if it does, writing secret keys securely for the CIS and host device in advance increases implementation costs. For this reason, this study uses the CIS-PUF [9,10], which extracts CMOS image sensor variations to generate responses, and random numbers to generate and share that key by utilizing the reverse fuzzy extractor (RFE) technique [11]. The PUF [12] is a technology that typically uses semiconductor manufacturing variations to generate device-specific responses, and the CIS-PUF, which applies the PUF technology to CISs, was proposed by our group [9]. In addition to the normal imaging mode, the CIS-PUF has two other modes of operation: PUF mode and random number generator (RNG) mode. The PUF mode generates device-specific responses from pixel variation information and is used for key generation. The random number generator mode extracts thermal noise from the CIS to generate random numbers, which are used for secure key sharing using RFE.

Image data captured by the CIS are sent to the host device through the MIPI, and the images are classified by an AI model implemented in a secure world. The MAC of the image data captured by the CIS is calculated in the CIS using the key generated from the CIS-PUF response. The MAC value and image data are then sent from the CIS to the host device. The key can be securely shared between the host device and the CIS using a random number (RN) generated by the CIS-PUF. The host device receives the image data in a secure world. MAC verification is performed using the shared key and the received image in a secure world. At this time, when the MAC verification is successful, image classification is performed using an AI model, and the classification results are output to the normal world. This system enables image tampering detection using the MAC, and the AI inference output of reliable MAC verification results is shared by the TEE.

**Figure 4:**
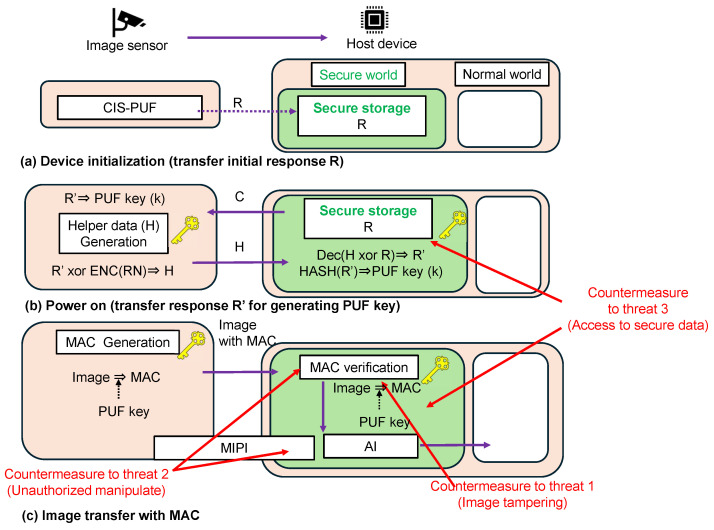
Block diagram and functions of the proposed countermeasure system.

Figure 3 is shown in greater detail in Figure 4. In advance, the initial response *R* of the CIS-PUF to a specific challenge *C* is stored in the secure storage in the TEE of the host device, as shown in Figure 4a. The data in the secure storage cannot be read or written from the normal world, which is a countermeasure against Threat 3. The RFE generates keys not from the initial response *R* but from the re-generated response $R^{\prime }$, which contains errors, and it shares the keys between the host device and the CIS for key generation. For the sharing of the secure response $R^{\prime }$, random numbers in RNG mode are used to create helper data, as shown in Figure 4b.

At power-on, the challenge *C* is input from the host device to generate a regenerated response $R^{\prime }$ in the PUF mode, and a PUF key *k* is generated from $R^{\prime }$. Since $R^{\prime }$ contains a response error, $R^{\prime }$ and *R* do not match, and a technique to securely share $R^{\prime }$ with the host device is necessary. By sending the XORed value (helper data) of the random number and *R* from the CIS-PUF to the host device, $R^{\prime }$ can be recovered from *R* using error correction techniques. The helper data are generated by encoding the random numbers generated in the RNG mode and XORing them with $R^{\prime }$. The helper data are sent from the CIS to the host device every time the power is turned on because the $R^{\prime }$ data disappear upon powering off. The host device reconstructs $R^{\prime }$ from the helper data and R, and it can generate the same PUF key generated in CIS. By inputting the shared $R^{\prime }$ data into the hash function, the same PUF key *k* is shared between the CIS and the host device.

As shown in Figure 4c, MAC verification is performed to check the authenticity of the captured images using the PUF key *k* in a secure world. Then, only when the MAC verification is authenticated, the image classification process is performed by the AI model. The AI classification process is also performed in a secure world, which is a countermeasure against Threat 3, such as tampering with or the theft of AI model parameters. The results of the inference after MAC verification are output to the normal world. From the MAC verification results, it is possible to detect whether the image data have been tampered with at the MIPI. Then, by processing in a secure world, measures such as skipping MAC verification can be taken, and reliable verification results can be output. The method of performing AI processing in a secure world with limited memory has already been discussed in other studies [13,14] and is beyond the scope of this study.

The countermeasures in Figure 4 correspond to the three threats shown in Figure 1. It is possible to add authenticity to an image, which is a countermeasure against Threat 1. The MIPI is connected to the image sensor via the secure world to prevent tampering with the image sensor, and MAC verification is performed in a secure world to obtain reliable results, which is a countermeasure against Threat 2. The ability to store the initial response R in secure storage in a TEE and the ability to prevent the leakage of AI model parameters are countermeasures against Threat 3.

In our implementation, the TEE is implemented using OP-TEE, which is an open-source TEE for the ARM processor. Since OP-TEE itself only provides an isolated execution environment, it is possible to implement a remote attestation that allows a third party to verify the authenticity of the executed software by performing a secure boot using the Root of Trust as the original operation. These discussions have been studied and verified for technology that implements a PUF in the host device and verifies it in a remote attestation [15,16]. Therefore, in this study, these techniques are out of the scope of implementation.

### 1.3. Our Contributions

The main contributions of this study are as follows:

- A system that adds a MAC to actual captured images using the PUF mode and RNG mode of our CIS-PUF test chip prototype is proposed and demonstrated.
- We proposed a method of calculating the MAC without storing the full image data on the CIS
- A system that processes MAC verification in a secure world of OP-TEE and performs secure MAC verification is demonstrated.

Our group previously proposed PUF response generation using CIS-PUF [9] and a random number generation method [10], and our novelty is that we proposed and implemented an image tampering detection system using these two methods in this study. In a previous study by other researchers, a perceptual HASH tampering detection system using CIS-PUF [17] was proposed [18]. However, that system was validated against a dataset that had already been captured by another image sensor. Our novelty is that the MAC was calculated in real time using the same CIS-PUF as that of the image captured by the image sensor, and the captured image and MAC were output simultaneously.

To implement the system shown in Figure 4, it is necessary to calculate the MAC in the CIS. However, there is not enough memory to store the full image data of one frame at once in the CIS, so the calculation of the MAC value with a small memory must be devised. We proposed a method of calculating the MAC value without storing the full image data on the CIS by sequentially inputting pixel data equivalent to the number of bits into the input block of the HASH function. The proposed method reduces the additional memory on the CIS from 3Mbits to 576bits, which is equivalent to one input block of the HASH function.

We demonstrated the system using a TEE so that MAC verification can be performed in a secure world. We experimentally confirmed that MAC verification was successful for normal capturing and that the MAC was mismatched when the image was tampered with.

## 2. System Components

**Figure 5:**
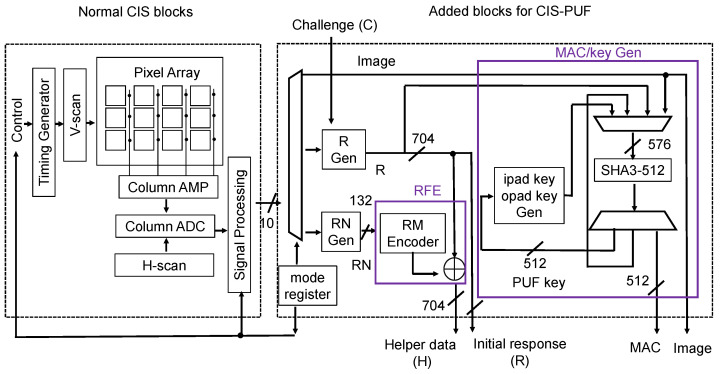
Block diagram of a CIS chip that generates a MAC using the CIS-PUF.

Figure 5 shows a block diagram of the CIS-PUF prototype composed of a test CIS chip and FPGA controller, which will be implemented on a CIS-PUF chip in the future to calculate the MAC from captured images. The CIS-PUF has the functions of the image sensor shown in Figure 4, and it outputs helper data, image data, and the MAC by operating in the image capture mode, PUF mode, and RNG mode.

First, the block diagram of a normal CIS shown in Figure 5 is explained. A timing generator controls the vertical scanner (V-scan) to drive the pixels. A mode control register switches the pixel operation mode to the imaging mode, PUF mode [9], and RNG mode [10]. Image acquisition is performed in the imaging mode; variations in each pixel are extracted to generate responses *R* in the PUF mode, and random noise is extracted to generate helper data in the RNG mode.

Next, the added block diagram for the CIS-PUF on the right side of Figure 5 is explained. The added block, which includes an RM (Reed–Muller) encoder for error correction and SHA3-512 for the hash function, performs processing to calculate the helper data and MAC value to be sent to the host device. In the PUF mode, the pixel to be used for the response is selected through challenge C, and the R generator (Gen) compares the output values from two adjacent pixels in the same column to generate responses. Then, the PUF response is input into SHA3-512 to generate a PUF key. In the RNG mode, the RN Gen extracts the least significant bit of the output value and performs XOR on two adjacent pixels in the same column to generate a random number. Then, helper data are generated according to the RFE mechanism. In imaging mode, an ipad key and opad key are generated from the PUF key, and the MAC is generated using these keys, the image data, and SHA3-512.

Section 2.1 explains the operation of the imaging mode, PUF mode, and RNG mode. Section 2.2 explains the RFE, which generates helper data to share the key generated from the response between the host device and CIS. Using the helper data, the host device can recover $R^{\prime }$ from the initial response R. Section 2.3 describes the calculation and implementation of the shared key and the MAC using SHA3-512.

### 2.1. CIS-PUF

**Figure 6:**
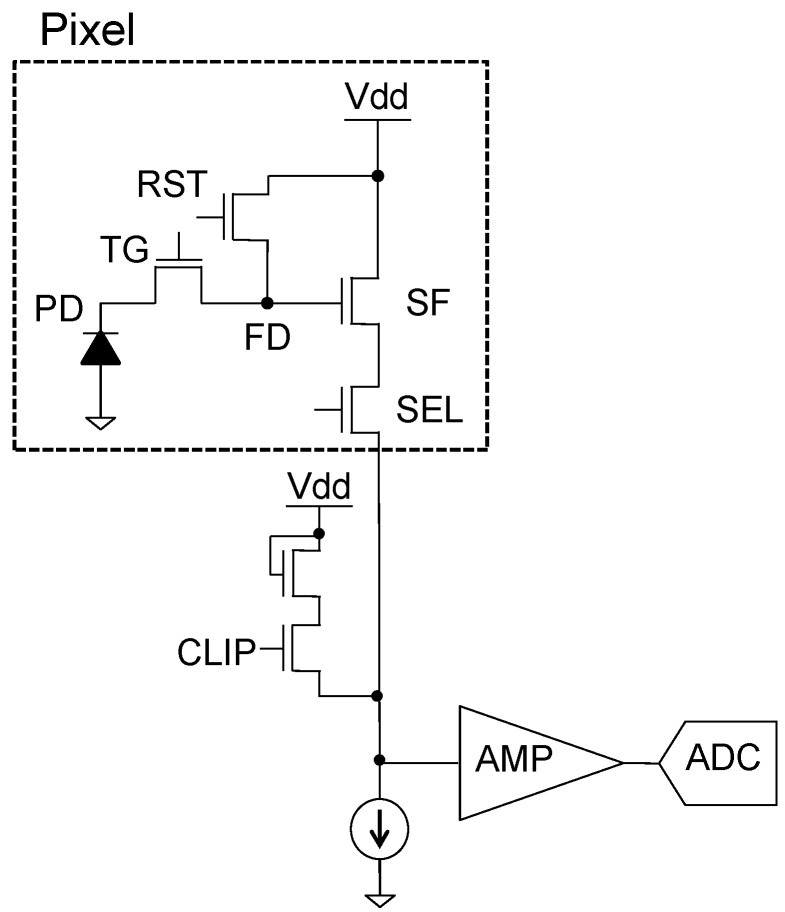
Readout circuit of the CIS.

**Figure 7:**
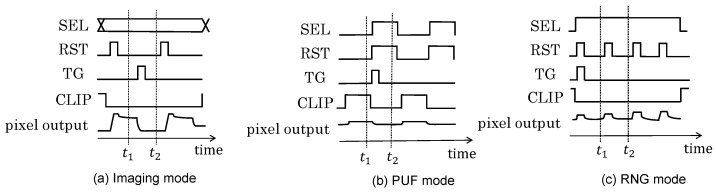
Readout timing for each mode.

Figure 6 shows the readout circuit of the CIS. A pixel is composed of a photodiode (PD) that performs photoelectric conversion, a floating diffusion layer (FD), a transfer gate (TG) that transfers the signal charge accumulated in the PD to the FD, a reset transistor (RST) that resets the potential of the FD, a source follower transistor (SF) that constitutes a source follower amplifier, and a selection transistor (SEL) that selects the pixel to be read out. A CLIP circuit is implemented for each column, and it supplies current so that the voltage does not drop when the SEL switches between rows when reading out image data. The signal readout timing for each mode is explained in Figure 7. Variations in the CIS include fixed-pattern noise that occurs at fixed positions in the circuit and is characteristic of each pixel and random noise that occurs randomly regardless of position. To eliminate these characteristic variations, the imaging mode involves correlated double sampling (CDS), which takes the difference between the reset potential and the signal potential for each pixel, as shown in Figure 7a.

In the PUF mode shown in Figure 7b, the offset caused by the variation in the SF Tr, which was removed in the imaging mode, is extracted as fixed-pattern noise by changing the signal acquisition timing so that CDS is not performed. Here, the reference signal in the PUF mode is the offset of the SF Tr in the CLIP circuit, and CLIP is turned on and read out at $t_{1}$. Then, the reset potential of the pixel is measured with the RST and TG turned on ($t_{2}$) so that the output does not change with the light intensity, and the offset variation in the SF Tr is output as fixed-pattern noise by taking the difference between the two potentials at $t_{1}$ and $t_{2}$. To generate a response from the output value in the PUF mode, the response is determined as 1 or 0 by comparing the values of the adjacent pixels above and below. By comparing the two pixels above and below, the variations in the column FPN caused by using a common CLIP circuit can be removed, and the bias of the response can be reduced [9]. The pixel to be used to generate the response is determined through challenge *C*.

In the RNG mode shown in Figure 7c, first, the RST and TG are turned on to remove the influence of electrons generated by the PD. The amount of charge present in the FD fluctuates due to the thermal motion of electrons, and the potential of the FD fluctuates around Vdd, reflecting its capacitance. This is thermal noise. By turning off the RST and TG, the flow of charge from the PD is blocked, the value of the thermal noise is fixed, and then the first output ($t_{1}$) is read out. By turning the RST on and off again, the value of the thermal noise fluctuates, and the second output ($t_{2}$) is read out. Finally, by taking the difference between the outputs read at times $t_{1}$ and $t_{2}$, fixed-pattern noise contained in the output variation is removed, and the component mainly consisting of thermal noise is output. To generate a random number from the output value in the random number mode, the lowest bit of each pixel is obtained. This is because the lowest bit is most affected by fluctuations in the output voltage value due to random noise. Since there is a bias in the bits in this state, the two pixels above and below are XORed to reduce the bias of 1/0; that bit is used as the random number to be output in the RNG mode [10].

**Figure 8:**
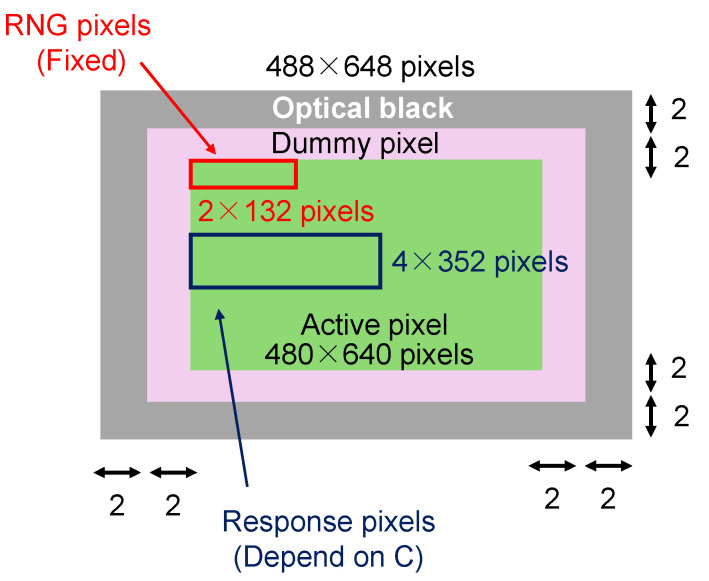
Pixel configuration of this implementation.

The pixel area that is actually used for image information is called the active pixel area, but there are also dummy pixel areas that are not used to obtain pixel information and optical black areas that determine the black level of the image. The pixel configuration in this implementation is shown in Figure 8. The total pixel size is 488 × 648, and the active pixel size is 480 × 640. The two pixels at the outermost edge are optical black pixels, and the next two pixels are dummy pixels. The response and random numbers are generated from the active pixel area. The PUF key *k* is shared using an RFE with the Reed–Muller code (32,6) (6 information bits, 32 codeword bits), so the response (*R* and $R^{\prime }$) is 704 bits, and the random numbers (RNs) are 132 bits. In the PUF mode, a 704-bit response is generated from the pixels in 4 rows × 352 columns specified by the challenge. In the RNG mode, a 132-bit random number is generated from the pixels in the fixed 2 rows × 132 columns on the top left.

### 2.2. Reverse Fuzzy Extractor

There are some error bits in the re-generated response $R^{\prime }$ because the PUF generates responses using minute physical variations. The fuzzy extractor (FE) [19] and reverse fuzzy extractor (RFE) [11], which use error-correcting codes, have been proposed as a countermeasure against errors.

Compared with the FE, the RFE does not require an error-correcting decoder circuit to be implemented on the PUF side, which reduces circuit costs. In this study, we used the RFE to reduce the circuit costs of the CIS. The RFE needs to share a regenerated response $R^{\prime }$ between the CIS-PUF and the host device, which is different from the initially registered response *R* stored on the host device. Therefore, it is necessary to generate helper data each time to share $R^{\prime }$ securely. Random numbers are required to generate helper data on the CIS-PUF, and the RNG mode of the CIS-PUF is used for the random numbers.

**Figure 9:**
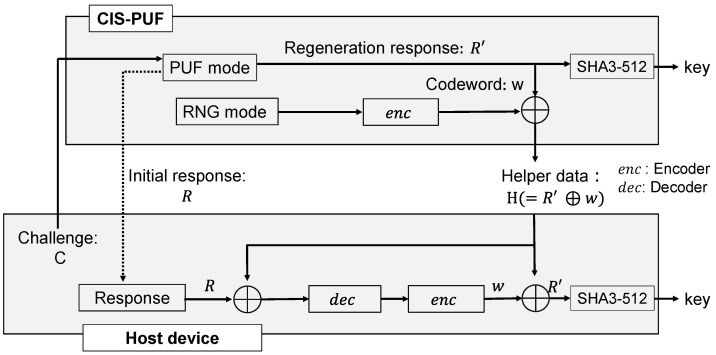
How to share keys using the reverse fuzzy extractor.

Figure 9 shows the key-sharing method using the RFE. First, the initial response *R* to a specific challenge is stored in the host device’s secure memory. Next, in order to generate a key in the PUF, the host device issues a challenge to the PUF and obtains the corresponding response $R^{\prime }$. Then, helper data *H* are calculated to generate the same key on the host device. Random numbers *x* are generated from the RNG mode, and the random number *x* is input into the encoder to obtain a code word *w*. Then, helper data *H* are created by XORing the code word *w* with the regenerated response $R^{\prime }$, and the helper data are sent to the host device. The helper data is public information that can be disclosed to the adversary because the response is masked with an encoded random number.

Next, a process is performed to generate a key on the host device that is the same as the key generated on the PUF side. The initial response *R* and the helper data *H* are XORed, and the resulting value is input into the error-correcting code decoder. The output from the decoder is input into the error-correcting code encoder to obtain the code word *C*. Then, the code word *w* obtained from the encoder is XORed with the helper data to obtain $R^{\prime }$, which is input into the hash function to regenerate the PUF key. When the error in the PUF response is below the error correction capability of the error-correcting code, the code word obtained from the encoder and the code word *w* generated by the PUF will be equal. As a result, the host device can generate a key that is the same as the key generated by the PUF.

Since the error rate of the PUF response of the CIS-PUF, which was evaluated while taking fluctuations in voltage and temperature into account, is 0.0162 [9], our implementation used RM(32,6) as the error correcting code. RM(32,6) is a Reed–Muller code with 6 information bits, 32 codeword bits, and 7 error-correctable bits. To create a key with 128 or more bits of information from an RFE using RM(32,6), 22 blocks are required (6×22=132>128). The helper data and required response are 704 bits (=32 × 22), and all bits of the response are input into SHA3-512 to generate the PUF key. The probability of an error correction failure in any of the 22 blocks, i.e., the key-sharing failure rate, was $7.76\times 10^{-7}$. This probability was smaller than the value of $10^{-6}$ defined in the previous literature [20] as the key generation failure index for FPGA implementations using a PUF.

### 2.3. Implementation of HMAC Using SHA3-512

In this implementation, SHA3-512 was used for MAC calculation and PUF key generation. Real-time MAC generation while reading image data is difficult with SHA-2, which is widely used today, because the throughput for hashing is low. For example, SHA2-512 requires twice as many clocks per input bit as SHA3-512. Therefore, to implement SHA2-512, additional memory to hold the image data or two SHA2-512 circuits must be implemented, which increases the circuit area. SHA-3 is used to calculate the MAC in the proposed system. SHA3-512 is selected because it has the worst throughput among the SHA3 types. When the calculations for SHA3-512 can be completed in time for the image read speed, it can be changed to other SHA3 series depending on the applications.

**Figure 10:**
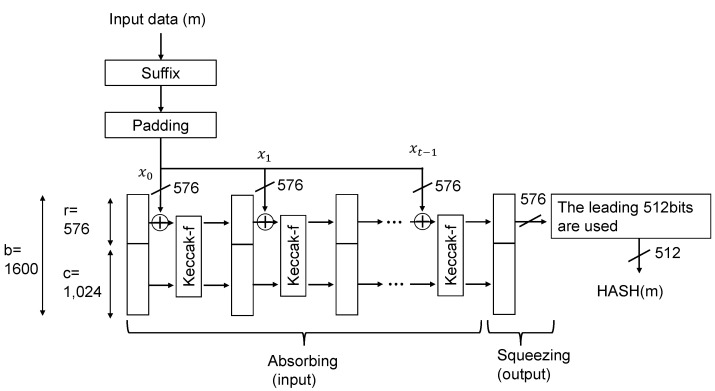
Block diagram of the hash calculation using SHA3-512.

Figure 10 shows a block diagram of the hash calculation using SHA3-512. SHA3-512 outputs a 512-bit HASH value for input data of any bit length. SHA3 calculates the HASH using the Keccak algorithm, which has an absorbing (input) phase and a squeezing (output) phase. The Keccak configuration parameters are b (state width) = 1600, r (block length) = 576, and c (capacity) = 1024. When data (m) are input into SHA3-512 in the input phase, they are divided into suffixes and input blocks through preprocessing. Padding is performed on blocks that are not sufficient to make up the 576 bits of the input block. As a result, the data are divided into t blocks, $x_{0}$ to $x_{t-1}$. As shown in Figure 10, the first block is input into the Keccak-f function for each block, and the next block is input after the Keccak-f function processing for that block is completed. This process continues until the last block, which is the input phase. Then, HASH(m) is output in the output phase.

A hash-function-based MAC is called HMAC, and we calculate the MAC using the HMAC with SHA3-512. The 704-bit response is input into SHA3-512 to generate a 512-bit PUF key *k*, and the HMAC is calculated from one frame of image data. First, an ipad key and an opad key are generated from the 512-bit key. Then, the ipad key and one frame of image data are input into the hash function to calculate the HASH value. After that, the concatenated value of the HASH value and the opad key is inputted into the HASH function to generate the MAC.

In this implementation, one HMAC is calculated from one frame of image data (640×480 pixels). If one frame of image data is to be input into the HASH function and all of the image data are stored in the CIS, a memory of 640×480×10 bits ≒3 Mbits is required in the CIS. However, implementing memory for one frame is unrealistic because it would result in an extreme increase in circuit area. We aim to save memory by calculating the HASH in accordance with the timing of the image data reading.

**Figure 11:**
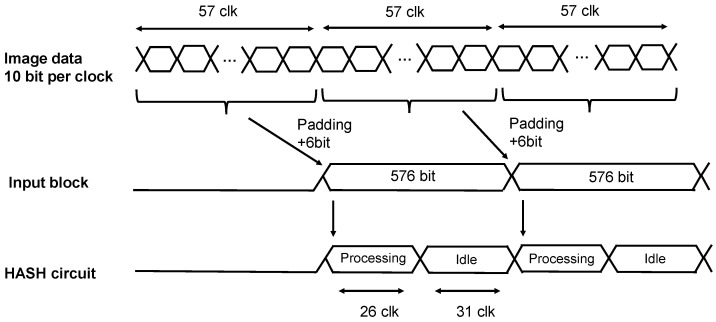
Timing of the HASH calculation for image data using SHA3-512.

Figure 11 shows the number of clocks required for one input block of the HASH function and the operation timing of the HASH circuit. Image data are read out in one clock for 10 bits per pixel. Since one input block of SHA3-512 is 576 bits, 57 pixels (570 bits) are padded with 6 bits to input one block into the HASH function. In the absorbing phase, it takes 26 clocks to process the Keccak-f function for one block of data. Therefore, there is a 26 (=57−31) clock idle state until the next data are input. By inputting each 570-bit block of pixel data into the HASH function in sequence, it is possible to reduce the additional memory required for MAC calculations from 3 Mbits to 576 bits.

## 3. System Configuration and Operations

### 3.1. Implementation of Experimental System

**Figure 12:**
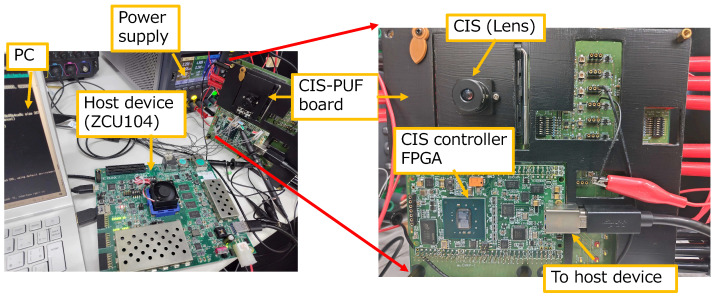
Experimental setup for the verification of the image tampering detection system.

The implementation setup is shown in Figure 12. The experimental setup consisted of a CIS-PUF board, an FPGA for the host device (Xilinx ZCU104), and a PC for controlling the host device. A customized CIS chip and FPGA (Humandata EDX-009-160T) as a CIS controller were implemented on the experimental CIS-PUF board. The CIS-PUF board included the circuit block depicted in Figure 5. The customized CIS was operated by supplying power from an external source and receiving control signals from the CIS controller’s FPGA. The three (imaging, PUF, and RNG) modes were controlled by changing various control signals with the timing shown in Figure 7. An additional circuit for the CIS-PUF shown in Figure 5 was implemented in hardware in the CIS controller’s FPGA outside the CIS. The CIS board included an ADC that converted one pixel’s output into a 10-bit digital value. The output from the ADC was then sent to the CIS controller’s FPGA. These circuits mounted on the CIS-PUF board are planned to be integrated into a CIS-PUF chip in the future.

**Figure 13:**
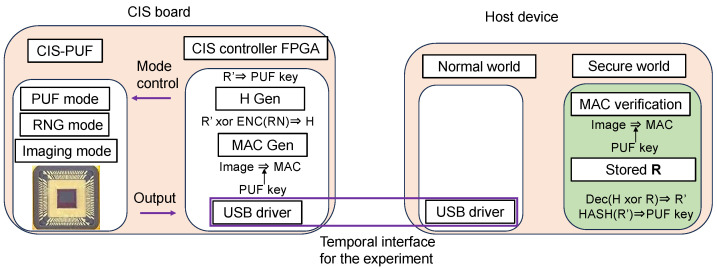
A diagram of the configuration of the implemented system.

The configuration of the implemented system is shown in Figure 13. The differences between the system implemented in this experiment and the proposed countermeasures shown in Figure 4 are described below.

- Instead of implementing a CIS chip to calculate helper data and MAC values, these processes were performed by the CIS controller’s FPGA. A CIS board with the CIS and FPGA performed the proposed CIS-PUF functions.
- Ideally, the CIS-PUF and host device were connected via a MIPI, but due to limitations of the board used, they were connected via USB in the normal world.
- Ideally, AI processing of captured images would be performed on the host device in a secure world, but AI processing was not performed in the experiment.

The host device had a normal world and secure world that was implemented with OP-TEE. By using the OP-TEE functions, it was possible to store image data and MAC values in secure memory and run applications in a secure world. All processes, such as MAC verification in the host device, were implemented in software in this implementation. The USB driver and CIS output operations are implemented in a Python 3.9, and the OP-TEE API and MAC verification are implemented in C language.

### 3.2. Device Initialization and Sharing Helper Data

As the device initialization process, the CIS-PUF response *R* was stored in the secure memory of the FPGA for the host device in advance.

**Figure 14:**
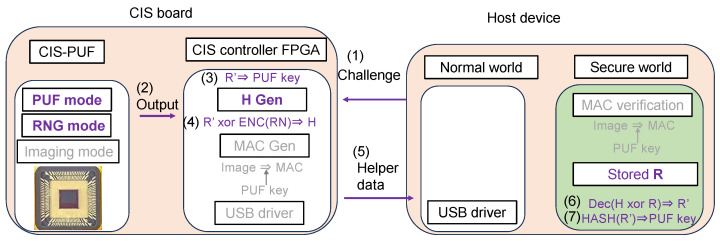
Helper data sharing operation (corresponding to Figure 4b).

PUF key generation and PUF key sharing using the RFE were performed as shown in Figure 14. The following processes were performed according to the order of the numbers shown in Figure 14.

(1) Challenge *C* was input from the host device into the CIS board. The CIS was operated once in the PUF mode and RNG mode, and raw data in the PUF mode and RNG mode were sent to the CIS controller’s FPGA.
(2) The outputs of the PUF mode and RNG mode were sent to the CIS FPGA controller to generate responses $R^{\prime }$ and a random number (RN).
(3) A PUF key was generated by inputting the PUF response into the HASH function.
(4) As with the PUF in Figure 9, helper data were calculated by XORing the response and encoded random numbers.
(5) The helper data *H* were sent to the FPGA for the host device.
(6) According to the RFE sequence shown in Figure 9, the regenerated response $R^{\prime }$ was restored using the saved initial response R and helper data.
(7) The same key was shared between the CIS-PUF and the host device by inputting $R^{\prime }$ into the HASH function to create the PUF key for the MAC.

### 3.3. Sending the MAC

**Figure 15:**
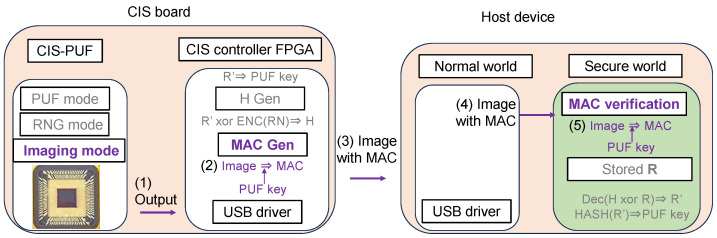
Operation in imaging mode (corresponding to Figure 4c).

When taking pictures in the imaging mode, the captured image was sent, and the MAC was calculated and verified as shown in Figure 15. The following processes are performed according to the order of the numbers shown in Figure 15.

(1) Raw data captured in the imaging mode were sent to the CIS controller’s FPGA.
(2) The CIS controller’s FPGA calculated the MAC using the PUF key and image data.
(3) The image data and MAC were then sent to the host device. Image data and MAC values were received from the CIS board through a USB interface in the normal world.
(4) These received data were sent to the secure world.
(5) In a secure world, the MAC was generated from the image data and PUF key, and the MAC was verified against the received MAC. The result of this MAC verification was sent to the normal world.

**Figure 16:**
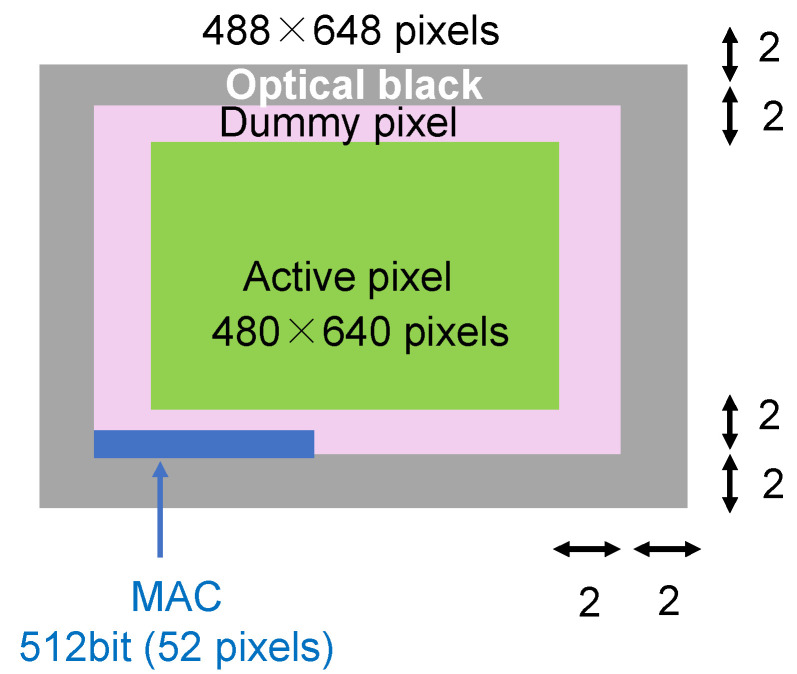
Data sent from the CIS board in the imaging mode.

The output data saved on the host device in the imaging mode included not only valid pixels but also dummy pixels and optical black pixels. The data format sent from the CIS board to the host device in the imaging mode is illustrated in Figure 16. Since the dummy pixels did not affect the image data even if the output value was not sent, the MAC value was sent from the CIS board by replacing the 512 bits (52 pixels) of the bottom row of the dummy pixels (486th row overall) with the MAC.

### 3.4. Operation Overhead

There is some data communication overhead, such as sending challenge data and helper data when the system is initially set up and when the device is started, as described in Section 3.2. However, when acquiring images as described in Section 3.3, MAC data are transmitted by using the timing when dummy data are sent in the conventional system. It is also noted that the size of MAC data is only 52 pixels, which is only 0.017% for the 480×640 pixels. Therefore, the impact of sending additional data on the overall system speed is not a problem in practical use.

## 4. Verification of the Image Tampering Detection System

**Figure 17:**
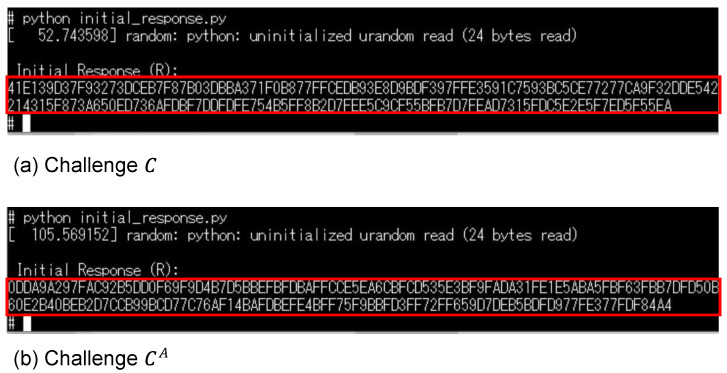
Initial response (704 bits) created by the PUF mode for two different challenges (*C* and $C^{A}$).

Our countermeasure system was verified using the experimental setup shown in Figure 12. First, the response *R* generated by inputting challenge *C* as the initial response was saved, and it corresponded to the device initialization shown in Figure 4. Challenge *C* used the PUF mode output values from lines 20 to 24. Figure 17a shows the result of displaying this initial response. Figure 17b shows the case where the input challenge was changed to $C^{A}$, which was different from *C*. Challenge $C^{A}$ used the PUF mode output values from lines 40 to 44. It can be seen that different responses were generated depending on the challenge.

**Figure 18:**
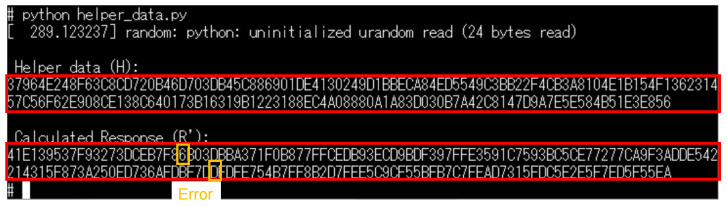
Helper data *H* obtained from the CIS board and the calculated response $R^{\prime }$.

In the operation shown in Figure 14, the PUF mode with challenge *C* and the random number mode were used, and helper data were sent from the CIS board. The result of displaying the helper data *H* is shown in Figure 18. It can be seen that the encoded random number masked the response, and the response was not revealed by the helper data. The regeneration response $R^{\prime }$ calculated from the helper data is also shown, and a response including errors (slightly different from the initial response) was generated.

**Figure 19:**
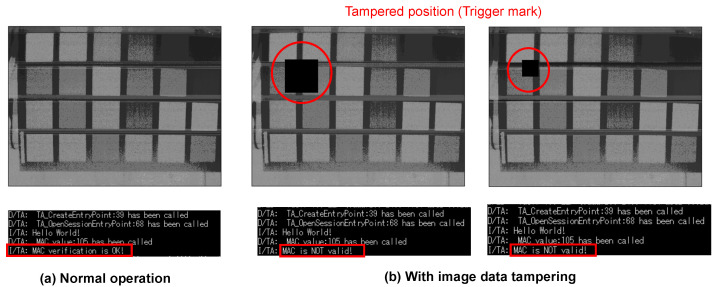
Verification results: (**a**) normal operation and (**b**) image data tampering.

The verification was performed by comparing normal capturing and tampered capturing. In the normal case, the same data used for the MAC calculation in the CIS were sent to the host device. In the tampered case, some pixel values of the image were intentionally tampered with in the CIS controller’s FPGA after calculating the MAC, and experiments were conducted with two different tampered sizes. In a real attack scenario, tampering is assumed to be performed through a fault injection attack on the MIPI. The captured images and verification results are shown in Figure 19. The verification results are displayed as they were obtained by the PC from the host device. It was confirmed that the MAC verification was successful when capturing normally but failed at both sizes when tampering was performed.

The tampering shown in Figure 19 simulated tampering that would trigger the backdoor shown in Figure 2. It was assumed that this captured image was input into a backdoor model that misclassified the image with a trigger mark in the top left. In this case, it was assumed that the captured image would be misclassified into the target class. However, the MAC verification was able to determine that the input image was tampered with, making it possible to detect the misclassification caused by image tampering through the fault injection attack.

**Figure 20:**
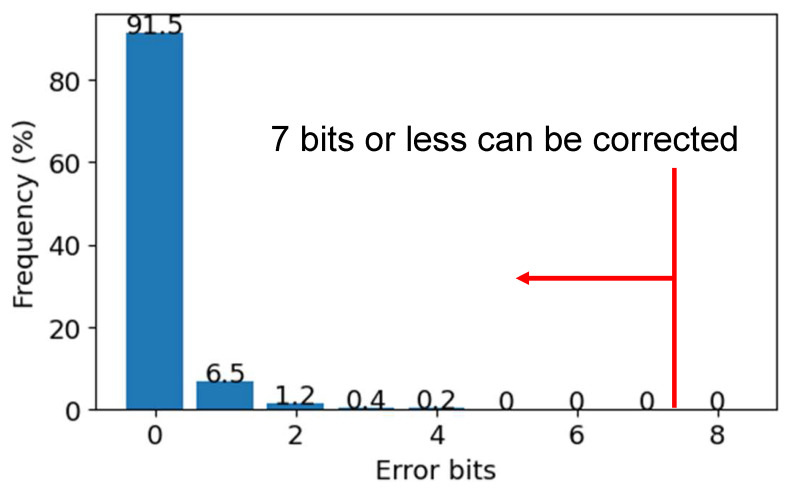
Frequency of error bits in one block on RM(32,6) code.

When considering the proposed system with respect to reliability and robustness, the most important issue is the successful key sharing between the image sensor and the host device. Key sharing requires the generation of a PUF response and error correction by RFE as described in Section 3.2. The response $R^{\prime }$ generated from the CIS-PUF at system startup has to be re-generated using R stored and the helper data *H* transmitted on the host device. The correction capability of the error correction code RM(32,6) used in RFE was experimentally evaluated. As described in Section 2.2, this code was determined from experimental results [9] evaluated in various environments. The 704-bit helper data H consists of 22 code words in 22 blocks. In the experiment, 120 different challenges were prepared for each block, and data were obtained on how many bits the 32-bit response $R^{\prime }$ differs from the initial response *R* when the same challenge was input to the PUF 50 times. The experimental results in Figure 20 show the number of error bits in each 32-bit block when $R^{\prime }$ is re-generated by a total of 22×120×50= 132,000 times. The largest error bit in this evaluation is 4 bits, and it can be successfully corrected since the error correcting code RM(32,6) can correct up to 7 bits out of 32 bits. Since these experimental results were obtained in a single evaluation environment, it is necessary to conduct evaluations in various environments for a sufficient discussion of reliability, which will be an issue to be addressed in the future.

## 5. Summary and Future Works

In this study, we proposed and demonstrated an image tampering detection system using a CIS-PUF and OP-TEE. The proposed system can counter threats of unauthorized operation and information leakage, as well as threats of tampering with image data transmitted by image sensors.

In the proposed tampering detection system, the PUF response and random numbers are generated from CIS-PUF, and a PUF key is generated from the PUF response. The RFE is used to share the PUF key with the host device, and the random number from the CIS-PUF is used to generate the helper data used for the RFE. The MAC is calculated using the HMAC with the PUF key for the image signal captured by the CIS, and authenticity is guaranteed by transmitting the image data with the MAC. Tampering can be detected by performing MAC verification in a secure world in a TEE.

The proposed system was demonstrated using a CIS-PUF board and FPGA as the host device. We succeeded in granting a MAC to the image data captured by the CIS and verifying this MAC. We confirmed that MAC authentication was successful when the image was taken normally but failed when part of the image was tampered with. In this experiment, our proposed system was evaluated against Threat 1 in Figure 1. In future work, we will need to evaluate against Threats 2 and 3. In addition, we plan to evaluate other physical attacks, such as side-channel attacks, to implement a more secure system.

The following elements in Figure 4 were not implemented in this demonstration, as described in Section 3. In future work, we will implement these elements and demonstrate the proposed system.

- This will be implemented on a CIS-PUF chip that includes the circuit block shown in Figure 5 to calculate helper data and the MAC.
- Communication between the CIS-PUF and the host device was performed via a MIPI, and this communication was performed in a secure world.
- AI processing of captured images will be performed in a secure world.

Another future challenge is the implementation of remote attestation, which would enable a third party to verify the authenticity of software by performing a secure boot using Root of Trust.

## Data Availability

The data are not publicly available. No data created for publication. If you have any concerns about the data, please contact the corresponding author.
